# *In situ* Microscopic Observation of Sodium Deposition/Dissolution on Sodium Electrode

**DOI:** 10.1038/srep22406

**Published:** 2016-03-01

**Authors:** Yuhki Yui, Masahiko Hayashi, Jiro Nakamura

**Affiliations:** 1 NTT Device Technology Labs., NTT Corporation 3-1, Morinosato Wakamiya, Atsugi-shi, Kanagawa Pref. 243-0198, Japan; 2 Department of Electronic Chemistry, Interdisciplinary Graduate School of Science and Engineering, Tokyo Institute of Technology, 4259 Nagatsuta, Midori-ku, Yokohama 226-8502, Japan.

## Abstract

Electrochemical sodium deposition/dissolution behaviors in propylene carbonate-based electrolyte solution were observed by means of *in situ* light microscopy. First, granular sodium was deposited at pits in a sodium electrode in the cathodic process. Then, the sodium particles grew linearly from the electrode surface, becoming needle-like in shape. In the subsequent anodic process, the sodium dissolved near the base of the needles on the sodium electrode and the so-called “*dead sodium*” broke away from the electrode. The mechanisms of electrochemical sodium deposition and dissolution on a copper electrode were similar to those on the sodium electrode.

Lithium-ion batteries are used for power sources in mobile devices and electric vehicles, and the demand for them is likely to increase. However, since lithium is not an abundant metal, it is expensive^1^. On the other hand, sodium is abundant and cheap, and interest in sodium-ion batteries (SIBs) has been growing^1^. Various materials have been studied for use as a SIB’s cathode or anode^2^,^3^,^4^,^5^,^6^,^7^,^8^,^9^,^10^,^11^. Most of these studies have been conducted on a half-cell system comprising a working electrode (WE), a counter electrode (CE), and if necessary, a reference electrode (RE). The WE contains the cathode or anode materials. In general, sodium metal sheets are used for the CE in the half-cell system. Using Na as the anode material is conceivable, but this would be difficult in practice because of safety issues.

There are many reports on electrochemical lithium deposition/dissolution^12^,^13^,^14^,^15^,^16^,^17^,^18^,^19^,^20^,^21^. Lithium metal electrodes have drawbacks of short circuiting and poor cyclability due to lithium “dendrite” formed during the electrochemical deposition. As reported in ref.^18^, the process of electrochemical lithium deposition in an electrolyte solution of LiAsF_2_/ethylene carbonate-2-methyltetra-hydrofuran at 0.5 mA/cm^2^ is as follows. Lithium grows from the base of the lithium electrode and creates kinks. Consequently, the shape of deposited lithium becomes dendritic. Then, lithium starts to deposit on the tip and at kink points of the lithium dendrite. The shape of the deposited lithium is particle-like. The process of electrochemical dissolution of the lithium dendrite is as follows^18^. The particle-like lithium on the tips and at kink points is dissolved. Then, the base of the dendrite is dissolved and the dendrite becomes *“dead lithium”*. This is a one of the causes of the poor reversibility of lithium metal electrodes. In addition, studies of the shape of electrochemically deposited lithium in various kinds of electrolyte have indicated electrolyte dependence of the shape^22^,^23^,^24^.

As describe above, there is accumulated scientific knowledge about electrochemical lithium deposition/dissolution. However, there are few reports^25^ on electrochemical sodium deposition/dissolution. It is important to understand behaviors, such as shape change during cycling, reversibility, and coulombic efficiency, to advance the development of SIBs. In this study, we focused on sodium metal as the CE and observed shape changes of electrochemically deposited/dissolved sodium in propylene carbonate (PC)-based electrolyte solution. The PC-based solution is widely used as the electrolyte for SIBs, and the use of a single solvent enables us to clarify the deposition/dissolution behaviors. Sodium and copper electrodes were used as WEs, and a sodium electrode was used as the CE as a typical reaction system. For observation, we used light microscopy as reported in our previous study on Sn-Co anode materials^10^. With this technique, we can observe cross-sections of the surface of sodium electrodes during the electrochemical sodium deposition/dissolution process and record video of sodium growth for *in situ* observation. In addition, to investigate whether surface thin layers are present on the deposited sodium like the solid electrolyte interphase (SEI), such as Li_2_CO_3_ and Li_2_O on deposited lithium^26^, we examined the surface composition of deposited sodium using a scanning electron microscope (SEM) equipped with an energy dispersive spectrometry (EDS) apparatus. Properties such as the ion conductivity of the SEI are important for achieving good cyclability.

## Results

### Observation of electrochemical sodium deposition on a sodium electrode in the cathodic process

**Figure 1:**
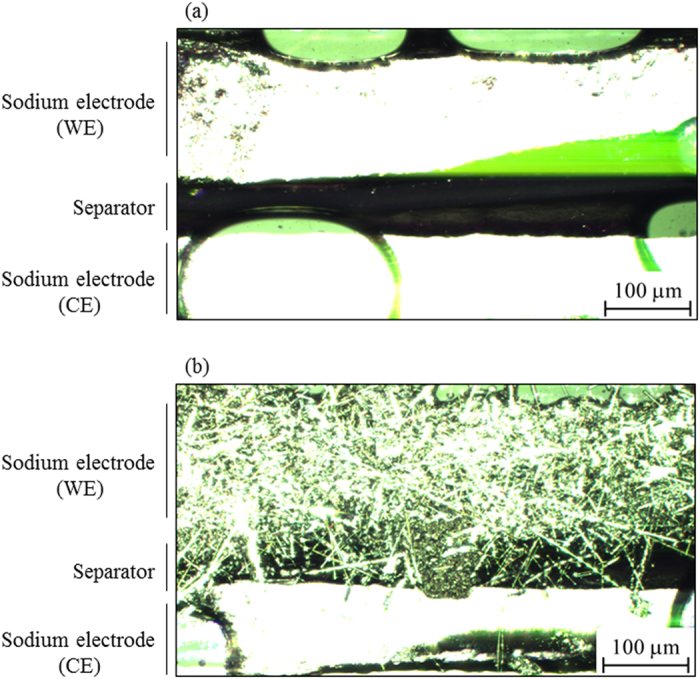
Cross-sectional views of a sodium electrode/separator/sodium electrode (**a**) before and (**b**) after sodium deposition of 300 μAh (after 360 min) at a constant current of 50 μA (56.6 μA/cm^2^).

The electrochemical sodium deposition/dissolution process was directly observed by using *in situ* light microscopy. Figure 1(a,b) show cross sections of the surface of the semicircular cell of a sodium electrode/1 M NaPF_6_/PC-soaked separator/sodium electrode before and after the cathodic sodium deposition process of 300 μAh (after 360 min), respectively (for video of the process, see Supplementary Movie 1). The small number of gas bubbles in Fig. 1(a) had entered at the time of cell preparation. However, we confirmed that they were not involved in the sodium deposition reaction due to argon gas. The pristine sodium electrode surface was relatively smooth; however, the electrode was densely covered with needle-like sodium after the deposition process as shown in Fig. 1(b). This image also suggests that the deposited needles grew not in a specific direction but in a random one. As will be described later in detail, the deposited product consists of multiple elements, not only sodium. However, in this paper, we use the term ‘sodium deposition’ for conciseness.

**Figure 2:**
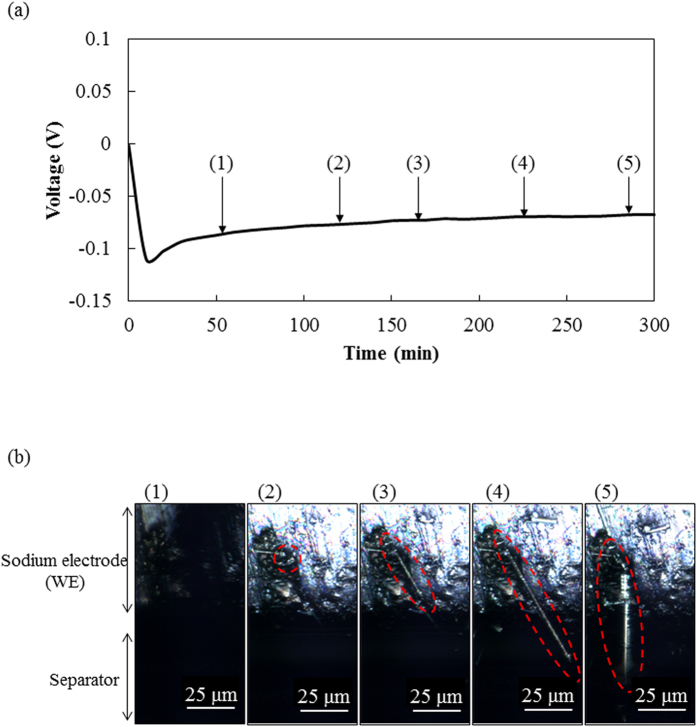
(**a**) Voltage change during electrochemical sodium deposition on a sodium electrode at a constant current of 50 μA (57 μA/cm^2^) and (**b**) light microscopy images of electrochemical sodium deposition on the sodium electrode in 1 M NaPF_6_/PC. The images were taken at sodium deposition of (1) 44.17 μAh (at 53 min), (2) 100 μAh (at 120 min), (3) 137.5 μAh (at 165 min), (4) 187.5 μAh (at 225 min) and (5) 237.5 μAh (at 285 min) in Fig. 2(a).

To clarify the growth mechanism of the needle-like deposition in more detail, we observed the semicircular cell during the deposition process at high magnification (for video of the process, see Supplementary Movie 2). Figure 2(a) shows the change in the voltage of the sodium electrode (WE) during electrochemical sodium deposition. The WE voltage dropped into the negative region against Na/Na^+^ (CE) voltage as soon as the deposition started, and the voltage transient gradually stabilized at around –0.06 V after 60 min. Figure 2(b) (1 shows light microscopy images of the sodium electrode (WE)/separator with the solution at the deposition time indicated by (1–5) in Fig. 2(a). The light microscopy image became dark due to gas evolution as shown in image (1). Next, sodium was deposited at a pit on a sodium electrode as shown in image (2). This deposition was regarded as granular nucleation for sodium growth. Then, the deposited sodium grew linearly from the sodium electrode surface, becoming needle-like in shape, and the needle-like sodium grew to a length of 36 μm as shown in images (3) and (4). The needle-like sodium swung from the base in an oblique to vertical direction as shown in image (5). The mechanism of electrochemical sodium deposition on a copper electrode was similar to that on the sodium electrode (see Supplementary Fig. 1).

### Observation of electrochemical sodium dissolution on a sodium electrode in the anodic process

**Figure 3:**
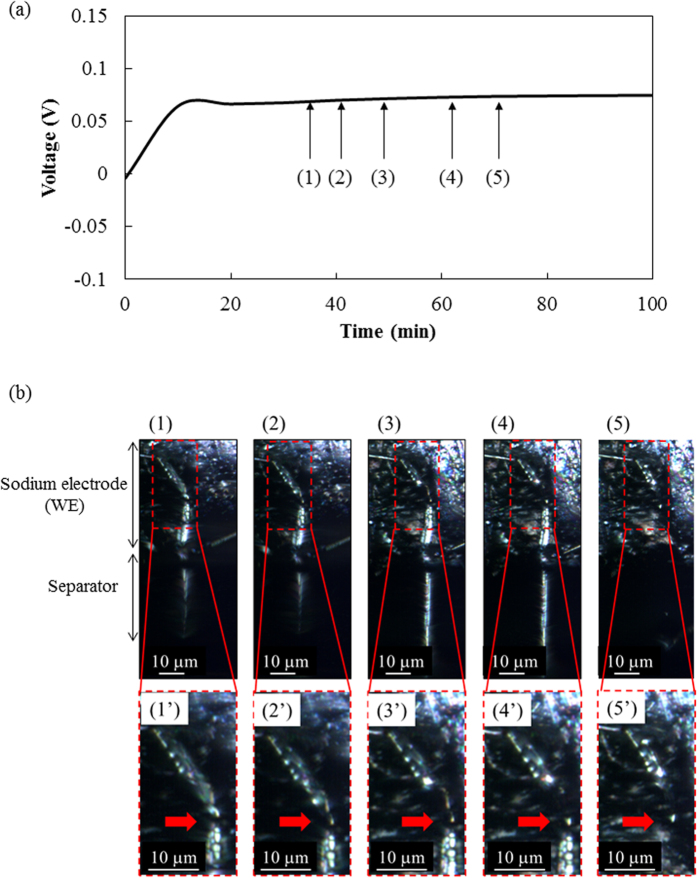
(**a**) Voltage change during electrochemical sodium dissolution on a sodium electrode at a constant current of 50 μA (57 μA/cm^2^) and (**b**) light microscopy images of electrochemical sodium dissolution on sodium electrode in 1 M NaPF_6_/PC. The images were taken at sodium dissolution of (1) 29.2 μAh (at 35 min), (2) 34.2 μAh (at 41 min), (3) 40.8 μAh (at 49 min), (4) 51.7 μAh (at 62 min) and (5) 59.2 μAh (at 71 min) in Fig. 3(a). Images (1’–5’) are microscopy images taken at higher magnification to observe the dissolution point.

Figure 3(a) shows the change in the voltage of a sodium electrode (WE) during anodic sodium dissolution. The voltage rose toward the positive direction against Na/Na^+^ (CE) voltage as soon as the dissolution started, and the voltage transient stabilized at around 0.06 V after 20 min. *In situ* light microscopy images of sodium dissolution on a sodium electrode are shown in Fig. 3(b) (1, 2, 3, 4, 5). These are images of the sodium electrode (WE)/separator soaked in electrolyte at the dissolution time indicated by (1–5) in Fig. 3(a). Figure 3(b) (1’–5’) are microscopy images taken at higher magnification to observe the dissolution point, indicated by red arrows, in more detail. The sodium dissolved near the base of a needle on the sodium electrode, and the needle became thinner as shown in images (1–4). Next, the needle-like sodium broke away from the sodium electrode as shown in image (5). That is, the needle-like sodium isolated from the electrode is regarded as so-called “*dead sodium*”. Similar behavior in dendrite/whisker lithium dissolution has been observed, indicating the formation of “*dead lithium*”^18^. The dead sodium would not be involved in the electrochemical reaction, afterward.

### Evaluation of coulombic efficiency with respect to sodium deposition/dissolution

Coulombic efficiency was evaluated with a 2032 coin-type cell using a copper sheet as a WE. The efficiency is defined as the percent ratio of the amount of electric quantity accumulated for dissolution to that for deposition. In addition, we also observed that the electrochemical sodium dissolution on a copper electrode was very similar to the process on the sodium electrode (see Supplementary Fig. 2).

**Figure 4:**
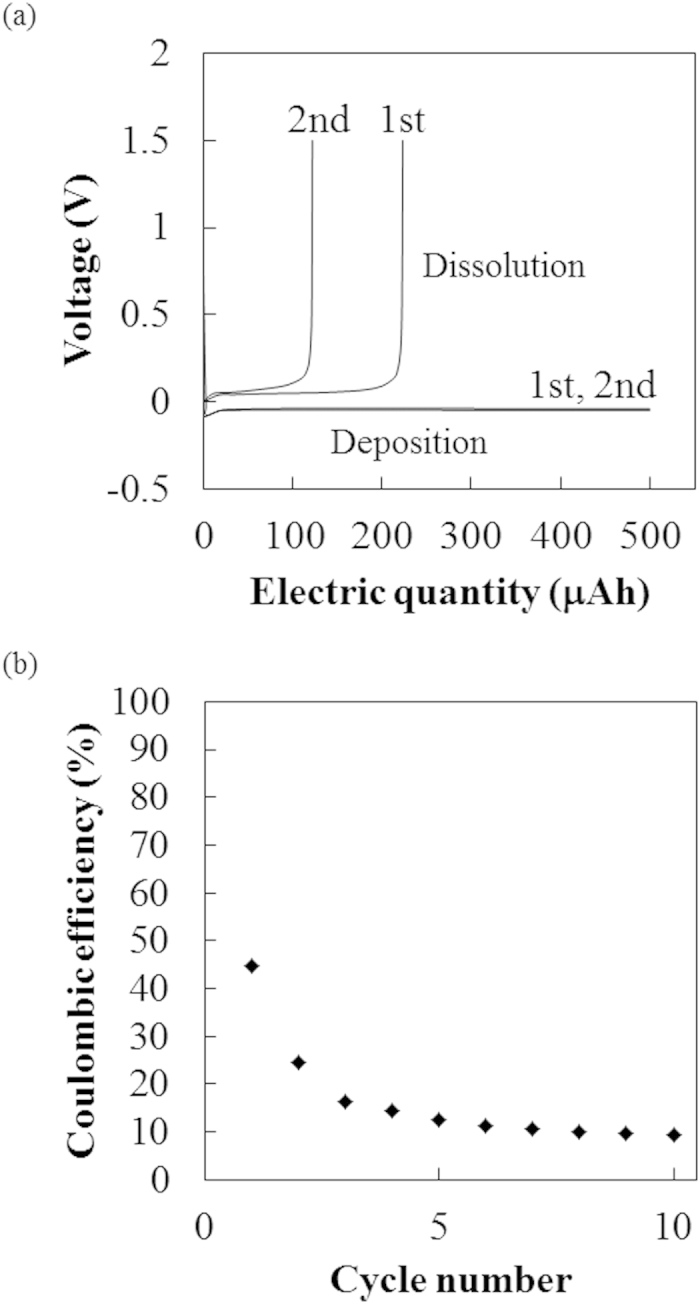
(**a**) Voltage profiles of sodium deposition/dissolution for evaluation of coulombic efficiency on a copper electrode in 1 M NaPF_6_/PC at a constant current of 100 μA (57 μA/cm^2^) and (**b**) efficiency changes with the number of cycles. The quantity of sodium deposition was 500 μAh in each cycle. The cutoff voltage for electrochemical dissolution was 1.5 V *vs.* Na/Na^+^.

The voltage profiles of sodium deposition/dissolution for evaluation of coulombic efficiency and the change in efficiency with the number of cycles are shown in Fig. 4(a,b), respectively. The efficiency was about 45% at the first cycle. The efficiency became lower with each cycle, with very low efficiency of less than 10% observed at the tenth cycle. Although experimental conditions, such as the solute of the electrolyte solution and current density are different, this efficiency for sodium is lower than that for lithium in PC electrolyte^21^. In ref.^21^, the efficiency for lithium was 60–80% during ten cycles. This suggests that the sodium electrode might not be suitable as a CE or a negative electrode because of the irreversible reactions. For this reason, we intensively investigated the composition of electrochemically deposited sodium using a copper electrode as the WE.

### Evaluation of composition of sodium deposition on a copper electrode

**Figure 5:**
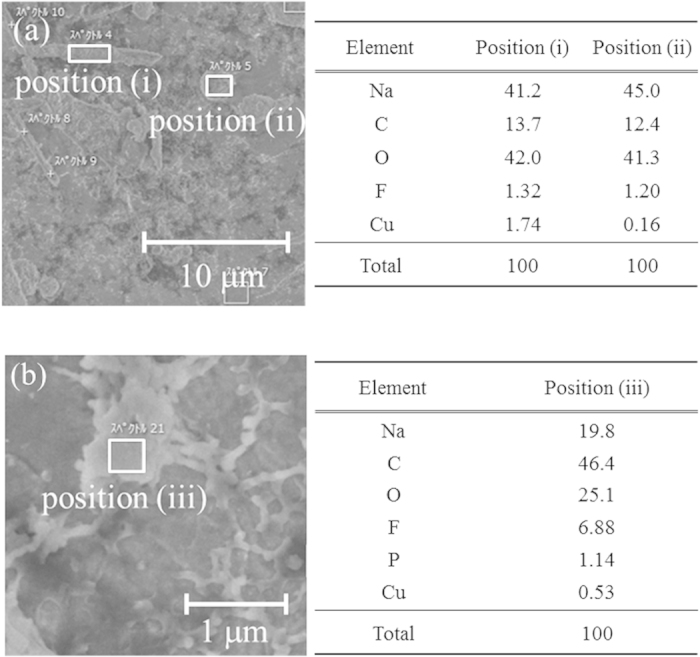
SEM images of electrochemically deposited sodium on a copper electrode and tables listing the element composition of the deposited sodium, detected by EDS in 1 M NaPF_6_/PC: (**a**) needle-like and granular morphology deposition and (**b**) film-like deposition.

Electrochemical sodium depositions on a copper electrode were analyzed by SEM-EDS to clarify the compositions of the deposited material. Figure 5 shows SEM images of a sodium-deposited copper electrode, together with tables listing the element composition of the deposited material detected by EDS. Figure 5(a) position (i) shows needle-like morphology of deposited material on the copper electrode, and the needle in this image is 10-μm long. EDS analysis revealed that the needle-like deposited material is composed of sodium, carbon, oxygen, and fluorine. In other words, the deposited material contained multiple components; it was not a pure phase of sodium. Figure 5(a) position (ii) shows deposited material with granular morphology on the copper electrode, and the diameter of the grains is about 0.2 μm. The morphology of this material might become needle-like thereafter. The EDS results for position (ii) show that the composition is the similar that at position (i). In addition, a mossy film can be seen in Fig. 5(b) at position (iii). Since the film is very thin, its morphology might not be confirmable by *in situ* light microscopy. EDS at position (iii) indicated a high content of carbon and fluorine.

## Discussion

**Figure 6:**
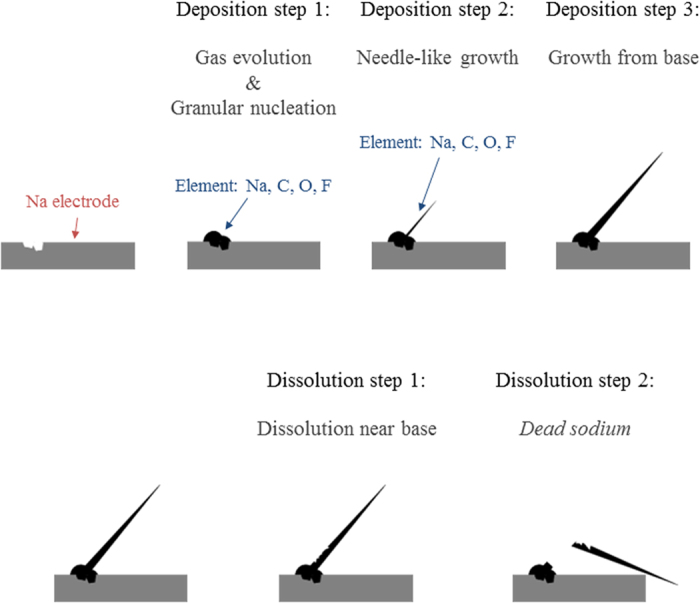
Pattern diagram of electrochemical sodium deposition and dissolution on copper electrode.

We observed electrochemical sodium deposition/dissolution reactions in PC-based electrolyte solution using *in situ* light microscopy. Figure 6 shows a pattern diagram of electrochemical sodium deposition/dissolution behavior on a sodium electrode. The process of sodium deposition follows three steps. First, the gas is produced, and granular sodium is deposited at pits on the electrode. The gas evolution is similar to that reported for lithium^26^. In ref.^26^, the decomposition of PC solvent in the presence of lithium was reported to result in the formation of propylene and Li_2_CO_3_. It is inferred from ref.^26^ that the generated gas was propylene derived from PC, and this indicates the possibility that Na_2_CO_3_ layers on the deposited sodium on a sodium electrode were formed by the decomposition of PC electrolyte accompanying the gas evolution. This would explain why the deposited material contained element species such as Na, C, and O. In addition, sodium is deposited on the pits because the electrical charge flow is concentrated at the edge of concave sites, where Na deposition proceeds more easily than elsewhere. Second, electrical charge flow is concentrated in the gap between the sodium particles, and compression stress is induced between them by Na deposition. Then, sodium particles grow linearly, becoming needle-like in shape. Third, the needle-like sodium grows further from the base.

In our ongoing experiments with 1 M NaPF_6_/EC:DEC 1:1 in volume as the electrolyte, needle-like sodium is observed to a greater or lesser extent (for video of the process, see Supplementary Movie 3). As reported in ref.^27^, the viscosities (*η*_0_) of PC, EC, and DEC solvent are 2.5, 1.9, and 0.75, respectively. Assuming that the additive property is satisfied, the viscosity of EC:DEC 1:1 in volume is 1.3, which is much lower than that of PC. That is, the viscosity of the solvent has little influence on the morphology of electrochemically deposited sodium. In addition, the permittivity (*ε*_r_) values of PC and EC:DEC 1:1 in volume (assuming that the additive property is satisfied) are 65 and 46. The large difference between these values suggests that the permittivity of solvent also has little influence on the morphology of electrochemically deposited sodium.

Additionally, the morphology of electrochemically deposited sodium is obviously different from that of lithium^18^. Deposited lithium creates kinks and its morphology is dendritic or whisker-like^18^; on the other hand, deposited sodium does not create kinks and its morphology is needle-like. The difference between sodium and lithium is possibly due to their different ionic mobilities at the interface of the electrode and electrolyte solution. Clarifying the origin of the needle-like sodium shape requires further investigation.

The process of electrochemical sodium dissolution follows two steps. First, the sodium is dissolved near the base of needles on the electrode. This is probably because the region near the tip of needles is nonconductor, which contains element species such as C, O, and F. The region near the base of needles is probably more conductive than near the tip since it is close to the metallic sodium electrode, and dissolution is likely to occur. Second, the so-called “*dead sodium*” breaks away from the electrode. This could depress the coulombic efficiency as shown in Fig. 4.

In addition, the deposited material contained element species such as Na, C, O, and F. It is highly possible that it contains Na_2_CO_3_, Na_2_O_x_ and NaF. These components are quite similar to those of the SEI on lithium metal in PC-based electrolyte solution^26^. The multicomponent depositions might be formed when the electrolyte decomposes. Unlike the SEI of lithium metal, because there was little sodium ion conductivity in the deposited material, the coulombic efficiency decreased. Furthermore, a mossy film was observed and its position had a high carbon and fluorine level. This suggests that it might be possible to control the morphology of deposited material by forming a suitable coating derived from the electrolyte. In other words, applying such additives to the electrolyte might improve of the irreversibility of the sodium electrode. Until irreversibility can be improved, it is necessary to take measures such as increasing the amount of the sodium metal. Hong *et al.*^28^ have recently shown good cycling stability even though they used sodium metal as a counter electrode. They therefore might have filled the cell with a large amount of sodium.

In conclusion, the behaviors of electrochemical sodium deposition and dissolution were almost independent from the type of electrode materials, such as sodium or copper. Granular sodium was deposited at pits on an electrode. Then, the sodium particles grew linearly from the electrode surface, becoming needle-like in shape. The process of dissolution is as follow. The sodium was dissolved near the base of the needles and became “*dead sodium*”. This would cause low coulombic efficiency with respect to sodium deposition/dissolution. As an overall result, we found that sodium has unsatisfactory irreversibility as the counter electrode for evaluating the electrode materials of SIBs. Such a low irreversibility would cause large amounts of sodium to be stored in the electrochemical cell.

## Methods

### *In situ* light microscopy

Change in morphology of a sodium or copper electrode during the electrochemical deposition/dissolution process were directly observed by using *in situ* light microscopy (Lasertec Corp., ECCS B310) in the same manner as reported previously^10^. The circular cell for the microscopy observation was prepared by stacking a sodium sheet (0.2-mm thick and 15 mm in diameter, Kanto Chemical Corp.) or a copper sheet (0.01-mm thick and 15 mm in diameter) as a WE, an electrolyte solution (1 M NaPF_6_/PC, Tomiyama Pure Chemicals Industries Ltd.)-soaked polypropylene separator (19 mm in diameter, Celgard), and a sodium sheet as a CE. The circular cell was cut into semicircles, and the exposed cross-sectional surface of a cell of the WE/separator with electrolyte solution/CE was prepared for the observation as shown in Supplementary Fig. 3(a). Then, the semicircular cell was placed in a jig for light microscopy and the cross-section of the surface was monitored *via* an observation window made of sapphire using light microscopy as shown in Supplementary Fig. 3 (b). The electrochemical measurements were performed using an automatic galvanostatic discharge-charge system (Hokuto Denko Corp., HJ1001SD8) at a constant current of 50 μA (57 μA/cm^2^) at room temperature.

### Characterizathion

The cells were opened after the electrochemical deposition on the copper sheet in the Ar-filled glove box. The sodium-deposited WE was washed with dimethylcarbonate (Tomiyama Pure Chemicals Industries Ltd.) and transferred to the SEM (JSM-6380LV, JEOL) equipped with EDS (X-MAX2, Oxford Instruments) using an Ar-filled transfer vessel in order to prevent degradation of the sample by air exposure. The surface composition of the deposited sodium was thoroughly measured using the SEM-EDS. The operating voltage for EDS was 3 kV.

### Coulombic efficiency

Coulombic efficiency was evaluated with a 2032 coin-type cell using a copper sheet as a WE, an electrolyte solution (1 M NaPF_6_/PC)-soaked separator, and sodium sheet as a CE. Coulombic efficiency measurements were performed using the above galvanostatic discharge-charge system at a constant cathodic/anodic current of 100 μA (57 μA/cm^2^) at room temperature in the manner reported in ref.^10^ as follows. Electrochemical sodium deposition and dissolution were repeatedly carried out in such a manner that the sodium equivalent to the electric quantity of 500 μAh was deposited and dissolved until the cut-off voltage of 1.5 V (*vs.* Na/Na^+^). All the cells were assembled in an Ar-filled glove box (dew point <–75 °C, O_2_ <0.1 ppm).

## Additional Information

**How to cite this article**: Yui, Y. *et al.* *In situ* Microscopic Observation of Sodium Deposition/Dissolution on Sodium Electrode. *Sci. Rep.* **6**, 22406; doi: 10.1038/srep22406 (2016).

## Supplementary Material

Supplementary Movie 1

Supplementary Movie 2

Supplementary Movie 3

Supplementary Information
