# Supplementary material for: In situ Microscopic Observation of Sodium Deposition/Dissolution on Sodium Electrode
Source: Sci Rep. 2016 Mar 1;6:22406. doi: 10.1038/srep22406 (PMC4772482; doi:10.1038/srep22406)
Supplement: Supplementary Information [file srep22406-s4.pdf]

In situ Microscopic Observation of Sodium Deposition/Dissolution on Sodium Electrode

Yuhki Yui[1,2], Masahiko Hayashi[1], Jiro Nakamura[1,2]

[1]NTT Device Technology Laboratories, NTT Corporation, 3-1 Morinosato Wakamiya, Atsugi, Kanagawa 243-0198, Japan

[2]Tokyo Institute of Technology, 4259, Nagatsuta-cho, Midori-ku, Yokohama, Kanagawa 226-8502, Japan

*E-mail: yui.yuhki@lab.ntt.co.jp

1. Supplementary Figures

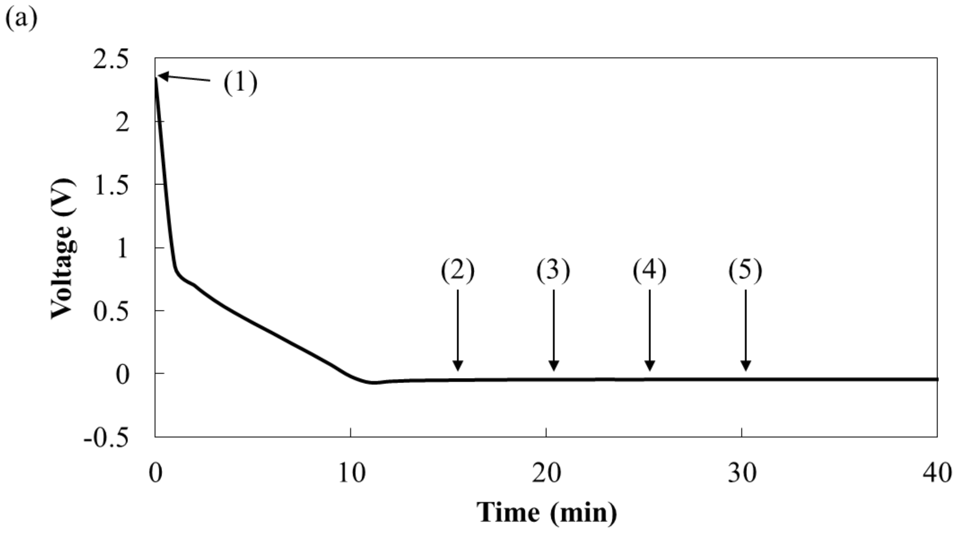

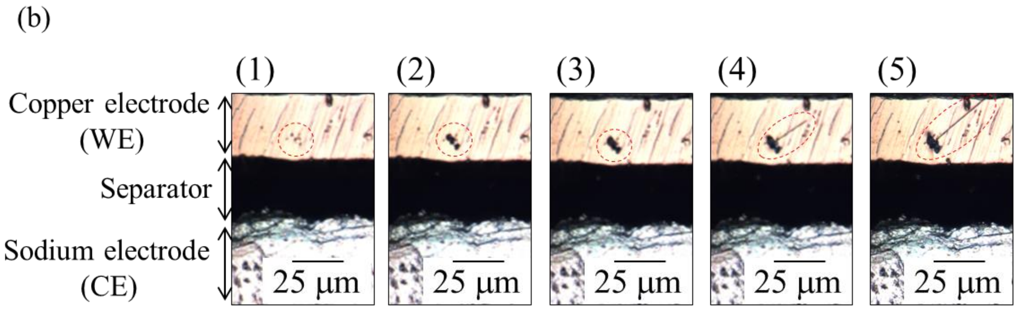

Supplementary Fig. 1 (a) Voltage change during electrochemical sodium dissolution on a copper electrode at a constant current of 50 μA (57 μA/cm^2) and (b) light microscopy images of sodium dissolution on copper electrode in 1M $NaPF_6$/PC, (1) sodium dissolution of 11.7 μAh (after 14 min), (2) 13.3 μAh (after 16 min), (3) 15.0 μAh (after 18 min), (4) 27.5 μAh (after 33 min), and (5) 28.3 μAh (after 34 min).

The voltage slowly decreased, and the voltage stabilized at around -0.05 V after 12 min. Image (1) is pristine cross section, and there were pits on the copper electrode in the red oval. As soon as the deposition started, the granular sodium was deposited at pits on a copper electrode in images (2)-(3). Then, sodium particles grew outward and the morphology changed to needle-like. This suggests that the electrochemical sodium deposition on a copper electrode was very similar to the processes on a sodium electrode.

(a)

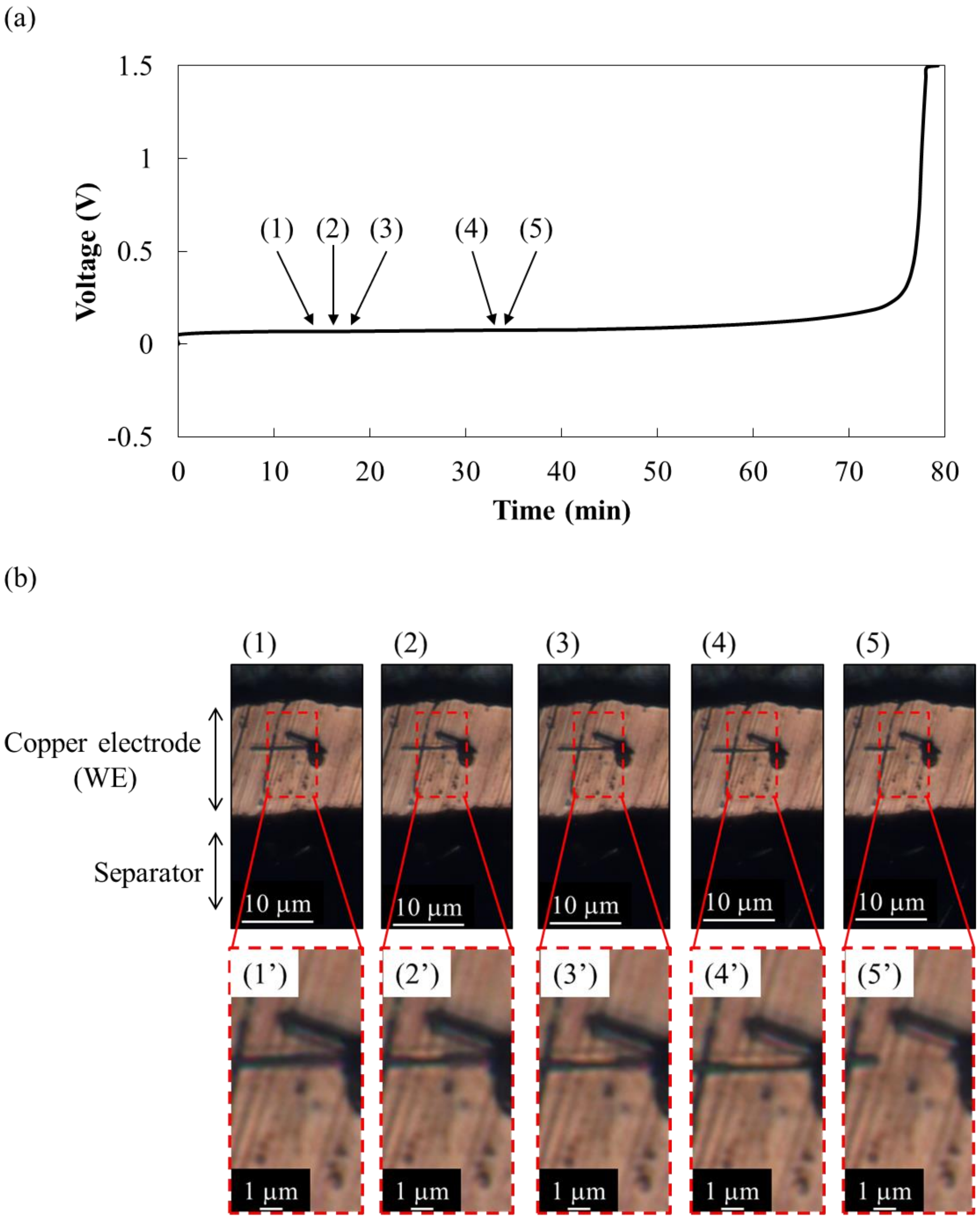

Supplementary Fig. 2 (a) Voltage change during electrochemical sodium deposition on a copper electrode and (b) light microscopy images of sodium deposition on a copper electrode in 1M $NaPF_6$/PC. (1) Pristine, (2) sodium deposition of 12.5 μAh (after 15 min), (3) 16.7μAh (after 20 min), (4) 20.8 μAh (after 25 min), and (5) 25.0 μAh (after 30 min). Images (1')-(5') are microscopy images taken at higher magnification to observe the dissolution point. The voltage stabilized at around 0.06 V as soon as the dissolution started. The sodium was dissolved near the base of the

needle on the copper electrode, and the needle became thinner as shown in images (1')-(4'). Then, the needle-like sodium broke away from a copper electrode as shown in image (5'). As has been noted, initial processes of sodium deposition such as nucleus formation were clarified by using copper as WE, and the mechanism of the electrochemical deposition and dissolution of sodium on copper electrode is similar to that for the sodium electrode.

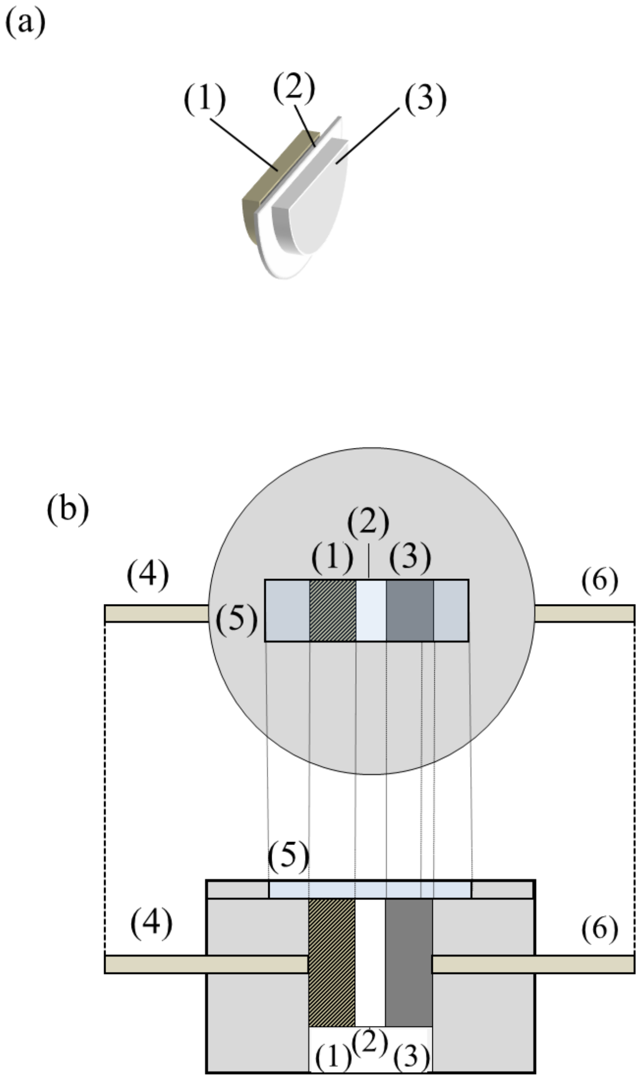

Supplementary Fig. 3 Schematic diagram of (a) the cell (overview) and (b) the jig (top and cross-sectional views) for *in situ* light microscopy: (1) WE, (2) separator

soaked with electrolyte solution, (3) CE, (4) WE terminal, (5) sapphire glass (observation window), (6) CE terminal.

2. Supplementary Movies

Movie 1: The video shows cross-sectional surfaces of the semicircular cell of a sodium electrode/1 M $NaPF_6$/PC-soaked separator/a sodium electrode during the sodium deposition process of 300 μAh at a constant current of 50 μA (57 μA/cm^2) at room temperature.

Movie 2: The video shows cross-sectional surfaces of the semicircular cell of a sodium electrode/1 M $NaPF_6$/PC-soaked separator/a sodium electrode at high magnification during the sodium deposition and dissolution process at a constant current of 50 μA (57 μA/cm^2) at room temperature.

Movie 3: The video shows cross-sectional surfaces of the semicircular cell of a sodium electrode/1 M $NaPF_6$/EC:DEC 1:1 in volume-soaked separator/a sodium electrode at high magnification during the sodium deposition and dissolution process at a constant current of 50 μA (57 μA/cm^2) at room temperature.
